# Ancient East Asian dog lineage is revealed by genome of ancient Korean dogs

**DOI:** 10.1371/journal.pone.0346864

**Published:** 2026-05-06

**Authors:** Hyeongcheol Kim, Suyeon Kim, A-reum Yu, Eunji Song, Sol Kim, Taiji Miyazaki, Yohey Terai

**Affiliations:** 1 Gaya National Research Institute of Cultural Heritage, Changwon, Republic of Korea; 2 Conservation Science Division, National Research Institute of Cultural Heritage, Daejeon, Republic of Korea; 3 Seokdang Museum of Dong-a University, Busan, Republic of Korea; 4 Foundation of East Asia Cultural Properties Institute, Changwon, Republic of Korea; 5 SOKENDAI (The Graduate University for Advanced Studies), Department of Evolutionary Studies of Biosystems, Hayama, Kanagawa, Japan; Københavns Universitet: Kobenhavns Universitet, DENMARK

## Abstract

Dogs form a monophyletic clade comprising three major sublineages: Eastern Eurasian, Western Eurasian, and sled dog lineages. While ancient genomic studies have clarified relationships within the latter two, the internal structure of the Eastern Eurasian lineage remains unclear. The dingo and New Guinea Singing Dog (NGSD) are regarded as its least admixed members, but it is uncertain whether they were the sole representatives in East Eurasia. Here, we sequenced ancient genomes from three dogs excavated at the Neuk-do site (3rd century BCE–early 1st century CE) in Sacheon and one dog from the Bonghwang-dong site (4th–6th century CE) in Gimhae. Average genomic coverages for the Neuk-do dogs were 0.5 × , 0.73 × , and 0.64 × , and 0.3× for the Bonghwang-dong dog. Phylogenetic analyses, outgroup-*f₃* statistics, and principal component analysis (PCA) of dogs and wolves showed that ancient Korean dogs were closely related to the dingo and NGSD. However, dog-only PCA separated ancient Korean dogs from dingo/NGSD, indicating a distinct lineage within Eastern Eurasia. The detection of Western Eurasian ancestry in the Neuk-do dogs demonstrates that admixture had begun before the 3rd century BCE. The higher proportion of this ancestry in the Bonghwang-dong dog suggests that additional admixture occurred by the 4th to 6th centuries CE. These findings reveal that ancient Korean dogs represent a separate Eastern Eurasian lineage that had already experienced Western Eurasian gene flow by the late first millennium BCE, providing new insights into the evolutionary history of dogs in Eastern Eurasia.

## Introduction

Dogs (*Canis lupus familiaris*) are believed to be one of the first domesticated animals. Their origins trace back to ancient times, with estimates suggesting that the lineage of dogs diverged from that of wolves (*Canis lupus*) approximately 20,000–40,000 years ago [1,2]. Whole-genome analyses have revealed that dogs form a monophyletic clade [3–6]. Moreover, this clade diverges from an internal lineage of East Asian wolves, indicating that the dog lineage likely originated in East Asia [6].

The monophyletic clade of dogs has separated into three major sublineages. Two of these are Eastern and Western Eurasian lineages [2,3,5–10]. The third lineage, sled dogs, consists of Arctic sled dogs and pre-European contact dogs of the Americas, including Siberian dogs and Greenland sled dogs [5,9,11–13]. Ancient genomic data from dogs have been reported [2,5,8,11,14,15]. Most of these ancient individuals belong to either the Western Eurasian lineage or the sled-dog lineage [2,5,8,11,14]. Consequently, some aspects of the internal relationships within each of these two lineages have been characterized. Although admixture between Eastern and Western Eurasian dog lineages has been reported, the internal relationship within the Eastern Eurasian lineage remains unresolved. In the Eastern Eurasian lineage, the dingo and the New Guinea Singing Dog (NGSD) show the least admixture with other dog lineages [4,6,16]. Therefore, they are regarded as having preserved ancestral genomic traits of early Eastern Eurasian dogs. However, little genomic information is available to show the relationship within the Eastern Eurasian lineage and how these lineages spread and evolved from East Eurasia to Oceania.

Two major wolf introgression events have been identified in the evolutionary history of dogs. The first event involved gene flow from the wolf related to the modern southwestern Eurasian population into the ancestry of early Near Eastern and African dogs, as demonstrated by ancient dog genomes [17]. The second event involved introgression from the ancestor of the Japanese wolf into the ancestor of Eastern Eurasian dogs [6]. However, it remains uncertain whether ancient Eastern Eurasian dogs experienced gene flow from other regional wolf lineages.

**Fig 1:**
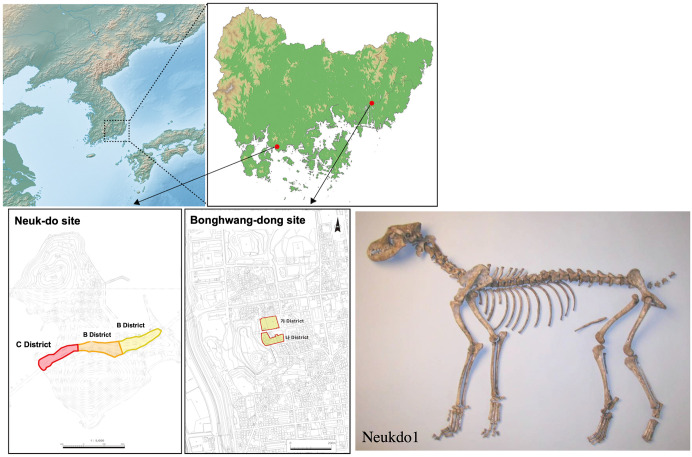
Map of the dog excavated sites with a photo of the skeleton of Neukdo1. In Neuk-do site, the dog bones were excavated from C District. In Bonghwang-dong site, the dog bone was exca- vated from the upper (northern) district. The base map of the Korean Peninsula was created using public domain data from the USGS EROS (http://eros.usgs.gov/). The map of Gyeongsangnam-do Province was produced using public domain GIS data provided by the National Geographic Information Institute of Korea (NGII), and all maps were edited using QGIS.

In Korea, dog remains have been excavated from archaeological sites dating back to the Neolithic period [18]. One notable site is Neuk-do, located on a small island off the southern coast of the Korean Peninsula (Fig 1). This site dates back to the 3rd century BCE and extends into the early Common Era (CE). At Neuk-do, numerous dog remains have been excavated [19,20]. Another site is Bonghwang-dong in Gimhae, which dates to the 4th and 6th centuries CE [21] (Fig 1). Dog bones were also excavated from this site. While the dogs found at these sites have been morphologically identified as small- to medium-sized individuals, their genetic relationship to other dogs remains unknown.

In this study, we sequenced ancient genomes from four dog specimens excavated from the Neuk-do site in Sacheon and the Bonghwang-dong site in Gimhae. By comparing these ancient Korean dog genomes with publicly available genomes of modern and ancient dogs and wolves, we clarified the genetic relationships between ancient Korean dogs and other canid lineages.

## Results

### DNA extraction and sequencing

We extracted DNA from three dog specimens excavated from the Neuk-do site (3rd century BCE to the beginning of CE) and one specimen from the Bonghwang-dong site (4th–6th centuries CE) (Fig 1). DNA extraction was performed using a minimally destructive method by dissolving the cementum layer of the tooth root. The extracted DNA was sequenced using the Illumina iSeq100 platform, producing tens of megabases of data for each sample. When these sequences were mapped to the dog reference genome (CanFam3.1), the estimated proportions of endogenous dog DNA in the extracts were as follows: 1.3% for dog No. 1 from the Neuk-do site (Neukdo1), 1.8% for dog No. 8 from the Neuk-do site (Neukdo8), 1.5% for dog No. 20 from the Neuk-do site (Neukdo20), and 0.8% for the Bonghwang-dong specimen (GB). We assessed the DNA damage patterns and reads from all four individuals showed the ancient DNA damage (S1 Fig).

We removed Uracil residues from the DNA extracts, and additionally constructed libraries and sequenced them using the Illumina NovaSeq X platform. We mapped the sequence reads to the dog reference genome (CanFam3.1). The average genomic coverages from each specimen were as follows: 0.5× for Neukdo1, 0.73× for Neukdo8, 0.64× for Neukdo20, and 0.3× for GB. We analyzed the ancient Korean dog genomes with the genome sequence data from a public database that contains 154 individuals, including six outgroup species, 36 wolves from North America and Eurasia including Korean two wolves [22], 30 ancient and 82 modern dogs [2,8,11,12,14,23,24] (see S1 Table). To explore the relationship between ancient Korean dogs and modern Korean dogs, our dataset includes the genomes of Donggyeongi, Jindo, and Sapsal dogs, as well as those of Korean wolves. Due to the low coverage of the ancient Korean dog data, we used a pseudo-haploid dataset for analyses.

### Genetic relationship of ancient Korean dogs to other canids

**Fig 2:**
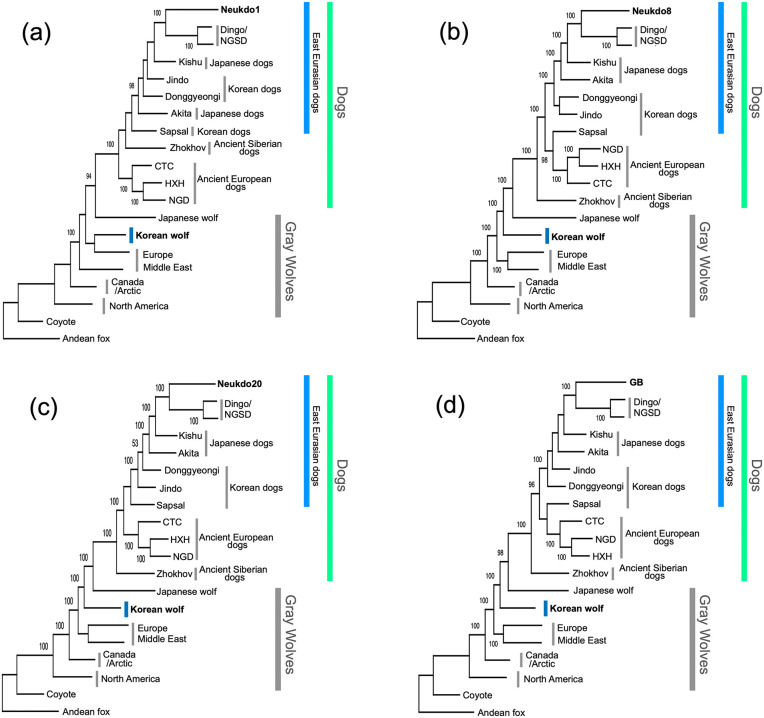
The ML trees of (a) Neukdo1, (b) Neukdo8, (c) Neukdo20, and (d) GB based on 14,517 sites for the Neukdo1, 196,547 sites for the Neukdo8, 221,326 sites for the Neukdo20, and 76,216 sites for the GB. The bootstrap values over 90 are shown at the nodes. Andean fox was used as an outgroup.

To explore the genetic relationships between ancient Korean dogs, other dogs, and wolves, we first constructed phylogenetic trees. Due to the limited number of overlapping SNP (single-nucleotide polymorphism) sites among the ancient Korean dogs (the number of sites was 423 in the dataset for PCA and Admixtools), we constructed four separate phylogenetic trees for each ancient Korean dog individual. The results indicated that all ancient Korean dogs belonged to the East Eurasian lineage and formed a monophyletic clade with the dingoes/NGSD (Fig 2). Furthermore, these ancient dogs showed a close genetic relationship with Japanese dogs such as the Akita and Kishu (Fig 2).

**Fig 3:**
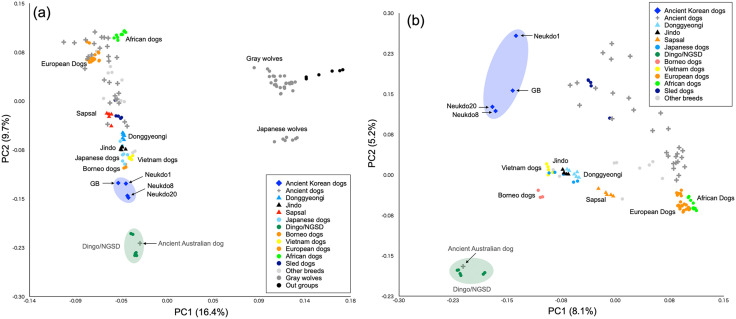
Relationships between ancient Korean dogs and other canids. Principal Components Analysis (PC1 versus PC2) of 158 ancient and modern dogs, wolves, outgroup species (a), and 116 dogs (b). Colored circles, squares, plus marks and triangles correspond to the names of dogs or wolves in the panel.

We explored the genetic relationships between ancient Korean dogs and other dog populations using principal component analysis (PCA). In the PCA based on ancient Korean dogs, other modern and ancient dogs, and wolves, the first principal component (PC1) separated dogs from wolves (Fig 3a). Meanwhile, the second principal component (PC2) formed a cline from Eastern to Western Eurasian dog populations (Fig 3a). Ancient Korean dogs cluster between dingoes/NGSDs and the dogs of Borneo, Vietnam, and Japan (Fig 3a).

**Fig 4:**
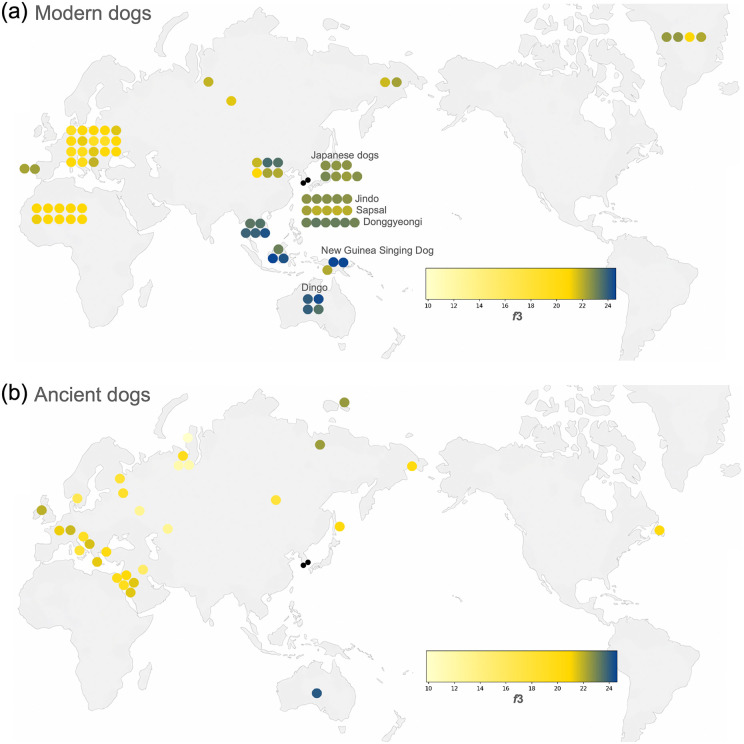
Shared genetic drift between dogs from Neukdo and Bonghwang-dong sites and all other modern dogs (a) and ancient dogs (b) measured by outgroup f3 statistics. Each circle represents the position of the origin of the dog, with the heatmap color indicating the f3 statistical value. The f3 values are shown in S2 Fig. Schematic world map created by the authors.

For the outgroup-*f3* statistics, we used four ancient Korean dogs as a population to increase the number of SNP sites shared between the ancient Korean dogs and the other ancient dogs. Outgroup-*f3* statistics revealed that the dingo, an ancient Australian dog, and NGSD showed the highest genetic affinity to ancient Korean dogs, followed by Bornean dogs and Vietnamese village dogs (Southeast Asian dogs) (Fig 4a and 4b, S2). Ancient Korean dogs have low genetic affinity with modern (Fig 4a, S2) and ancient (Fig 4b, S2) dogs in Western Eurasian and sled dog lineages.

Phylogenetic trees, outgroup-*f₃* statistics, and PCA using samples from both dogs and wolves indicate that ancient Korean dogs are closely related to the dingo and the NGSD (Figs 2, 3, and 4a). To further explore the relationships among East Eurasian dogs, we conducted a PCA specifically using only dog samples (Fig 3b). In this PCA focused on dogs, the first principal component (PC1) separated Eastern and Western dog lineages, while the second principal component (PC2) distinguished Northern and Southern populations within East Eurasia (Fig 3b). The dingo and NGSD formed a cluster that was farther from ancient Korean dogs and closer to Southeast Asian dogs, such as Vietnamese village dogs and Bornean dogs. In contrast, the ancient Korean dog cluster positioned the upper part of the PCA. These findings suggest that ancient Korean dogs and the dingo/NGSD are likely different lineages.

### Gene flow between ancient Korean dogs and other canids

Gene flow between dogs and wolves has been reported in previous studies [3,4,6,7,11,17,25–28]. However, the gene flow between ancient East Asian dogs and wolves has not been investigated. To explore potential gene flow between ancient Korean dogs and wolves, we analyzed it using *f₄* statistics. The results of the *f₄* statistics revealed that ancient Korean dogs exhibited the strongest gene flow with the Japanese wolf, followed by detectable gene flow with East Asian wolves from Korea and China (S3–S6 Figs). These findings are consistent with a previous study of gene flow between Japanese wolves and East Eurasian dogs [6]. These results indicate that ancient Korean dogs experienced gene flow with East Asian wolves. The gene flow between ancient Korean dogs and East Eurasian wolves did not specifically occur with Korean wolves, but instead it occurred with some East Asian wolf populations.

**Fig 5:**
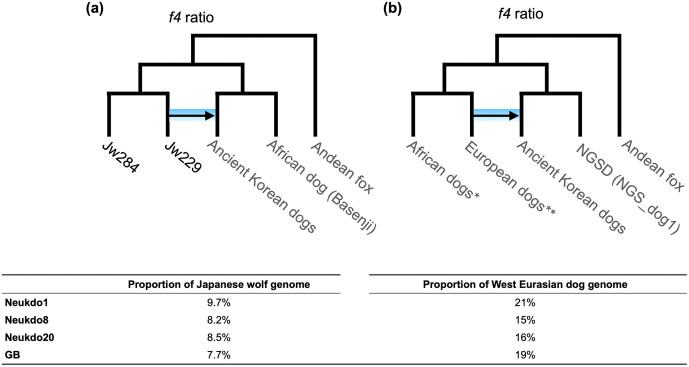
Proportion of (a) Japanese wolf ancestry and (b) European dog ancestry. *f4*-ratio (α) values of Japanese wolf ancestry for all dogs and European dog ancestry for East Eurasian dogs are shown in S7 and S9 Figs. All proportion values were supported by Z score > 3. *African_Dog1, African_Dog2, African_Dog3, African_Dog4, African_Dog5, Basenji, Nigerian_Indigenous_Dog1, Nigerian_Indigenous_Dog2, Nigerian_Indigenous_Dog3, and Nigerian_Indigenous_Dog4 were used as a African dog population. **Airedale_Terrier, American_Sta_Terrie, Boston_Terrier, Doberman_Pinscher, German_shepherd, Labrador_retriever1, Labrador_retriever2, Labrador_retriever3, Maltease, Miniature_Schnauzer, Portugal_Village_Dog1, Portugal_Village_Dog2, Scottish_Deerhounds, Standard_Poodle1, Standard_Poodle2, Yorkshire_Terrier were used as a European dog population.

A previous study indicated that the genomes of East Eurasian dogs contain the genetic ancestry of the Japanese wolf [6]. To evaluate this ancestry in ancient Korean dogs, we estimated the proportion of Japanese wolf ancestry using the *f₄*-ratio statistics. Our results showed that ancient Korean dogs harbored 7.5% to 9.7% Japanese wolf ancestry, a proportion comparable to that found in dingoes and NGSD, which ranged from 6.4% to 8.5% (Fig 5a, S7 Fig).

**Fig 6:**
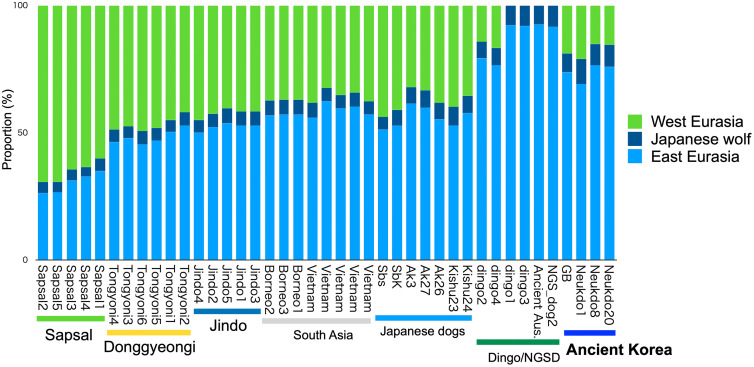
Proportion of ancestries of dogs in East Eurasian lineage. The East Eurasian ancestry was inferred as the remaining proportion of the genome after accounting for contributions from West Eurasian dogs and Japanese wolves. The proportion values of West Eurasia (European) dog ancestry and Japanese wolf ancestry are from S7 and S9 Figs.

Modern East Eurasian dogs have genetic components that originated from Western Eurasian lineage dogs [6,12,14]. To determine if this Western Eurasian ancestry was present in ancient Korean dogs, we used the *f₄* statistics and *f₄*-ratio. The *f₄* statistics showed the gene flow between ancient Korean dogs and Western Eurasian dogs (S8 Fig). The *f₄*-ratio revealed that 15–19% of the genomes of ancient Korean dogs were derived from Western Eurasian dog lineage (Figs 5b, 6, S9 Fig). Specifically, dogs from the older Neuk-do site, Neukdo1, Neukdo8, and Neukdo20, exhibited 21, 15, 16% Western Eurasian ancestry, respectively, while a more recent specimen from the Bonghwang-dong site (GB) showed 19% ancestry. The genomes of modern Korean dog breeds exhibit varying proportions of Western Eurasian ancestry, ranging from 41% to 45% in Jindo, 42% to 50% in Donggyeongi, and 60% to 70% in Sapsal. This variation indicates that the proportion of Western Eurasian ancestry in East Eurasian dogs has increased over time.

## Discussion

### A dog lineage from mid-latitude East Asia

The dingo and the NGSD are considered key representatives of the least admixed East Eurasian dog lineages and are used as representatives of East Eurasian dogs in genetic studies [6,14]. However, it is still uncertain whether they were the only dog lineages present in East Eurasia or if other distinct lineages also existed.

The PCA based on dog-only data in this study clearly separated ancient Korean dogs and dingo/NGSD into distinct clusters. The dingo/NGSD cluster was located close to that of southern populations, such as Vietnamese village dogs and Bornean dogs, indicating that they belong to a southern East Eurasian lineage. In contrast, the ancient Korean dog cluster is positioned in the upper part of the PCA, indicating that it represents a distinct lineage separate from the dingoes/NGSD cluster. These findings imply that at least two distinct dog lineages existed in East Eurasia.

Phylogenetic analysis, *f₃* statistics, and PCA with wolves, all indicated that ancient Korean dogs are genetically high affinity with the dingo and the NGSD. In contrast, most modern dog breeds, excluding the dingo and the NGSD, show proportions of Western Eurasian ancestry of at least 32% (Akita3). The observed genetic relationship between ancient Korean dogs and the dingoes/NGSD may be attributed to their relatively low levels of Western Eurasian introgression.

To better understand the internal genetic structure of East Eurasian dogs, it is crucial to analyze individuals that have minimal Western Eurasian ancestry, specifically ancient dogs. This study shed light on the two East Eurasian lineages, southern and mid-latitude, which was made possible by including ancient dogs in the analysis.

### Ancestries of ancient Korean dogs

The ancient dog from the Bonghwang-dong site exhibited a higher proportion of Western Eurasian ancestry in its genome, approximately 2–3% more than that found in dogs from the older Neuk-do site, Neukdo8 and Neukdo20 (the standard error of Neukdo1 was too high to compare). Although our analysis focused on a single individual from the Bonghwang-dong site, this difference likely reflects the chronological gap between the two archaeological contexts. The Neuk-do site dates from the 3rd century BCE to the beginning of the CE, while the Bonghwang-dong site dates from the 4th to the 6th centuries CE.

The presence of Western Eurasian ancestry in dogs from the earlier Neuk-do site suggests that admixture with Western Eurasian dogs had already occurred before the 3rd century BCE. The higher proportion observed in the 4th to 6th-century dog from Bonghwang-dong indicates that this admixture likely continued during that period. Given that modern East Asian dogs carry at least 32% western Eurasian ancestry, it is reasonable to conclude that increasing contact and exchange between Eastern and Western Eurasia over time contributed to a gradual rise in Western Eurasian genetic influence in East Eurasian dog populations.

A previous study has indicated that the Japanese wolf is most closely related to the domestic dog lineage and that there has been no gene flow between the Japanese wolf and Eurasian wolves [6]. Ancient Korean dogs also carried the ancestry of the Japanese wolf. The proportion of this ancestry is comparable to that seen in dingoes and NGSD, which may explain the genetic similarities shown in phylogenetic and *f₃* statistical analyses. Although the Japanese wolf existed on the Japanese archipelago until about a century ago [29], the gene flow between the Japanese wolf ancestor and an East Eurasian dog ancestor likely occurred on the East Asian mainland [6]. The presence of Japanese wolf ancestry in ancient Korean dog genomes indicates that this admixture occurred at least 2,000 years ago on the continent.

### Relationship of ancient and modern Korean canids

This study demonstrated that ancient Korean dogs experienced gene flow with East Asian wolves. However, the signature of introgression was not particularly strong with Korean wolves, suggesting that ancient Korean dogs likely did not frequently introgress with local Korean wolf populations.

In Korea, several indigenous dog breeds, such as the Jindo, Donggyeongi, and Sapsal, are being bred today. In our study, we were unable to show a direct genetic relationship (i.e., phylogenetic relationship) between ancient Korean dogs and these modern breeds. This lack of connection is likely due to the significant proportion of Western Eurasian ancestry found in modern Korean dogs.

In this study, we identified an East Asian dog lineage separate from dingoes/NGSD by examining the genomes of ancient Korean dogs and their genetic relationships with other dogs and wolves. The ancestors of ancient Korean dogs had already mixed with western Eurasian dogs at least 2,300 years ago. This admixture increased by the 4th to 6th centuries CE. In modern Korean dogs, approximately half of the genome is now derived from Western Eurasian ancestry. To further clarify the ancestry of ancient Korean dogs, it is essential to analyze the genomes of ancient Neolithic-era dogs in Korea. Such data will provide deeper insights into the evolutionary history of dogs in East Asia.

## Methods

### Sample information

We used the dog skeletal remains excavated from two archaeological sites in the southern Korean Peninsula: the Neuk-do site in Sacheon and the Bonghwang-dong site in Gimhae (S2 Table).

The Neuk-do site is a complex archaeological locality that encompasses an entire small island on the southern coast of Korea, spanning from the 3rd century BCE to around the CE (S3 Table). Excavations have revealed shell middens, burials, dwellings, and other features, along with a wide range of artifacts reflecting interactions with contemporary cultures in China and Japan. These findings suggest that the island functioned as a key hub for international exchange during this period [19,20,30]. Three dog bone specimens from Neuk-do, excavated by the Museum of Dong-A University, were selected for analysis. We analyzed the specimens with the permission and oversight of the curating institution.

The Bonghwang-dong site, located in Gimhae, South Gyeongsang Province, represents a major residential and ceremonial center of the Geumgwan Gaya polity during the 4th–6th centuries CE (S3 Table). The site, located south of the UNESCO World Heritage-listed Daeseong-dong tumuli, has yielded shell middens, stilted buildings, docking facilities, earthen fortifications, and large residential structures [21]. We included one dog bone specimen from an area currently under excavation by the National Research Institute of Gaya Heritage in this study. We conducted all analyses under the institute’s internal regulations governing the sampling and study of curated archaeological materials.

#### Ancient DNA extraction.

We extracted ancient DNA in the clean room specifically designed for ancient DNA analysis at Daejeon and Sokendai, using a modified protocol from Harney et al. [31]. Before sampling, all the tooth roots were wiped with 70% EtOH to remove surface contaminants.

We first soaked the tooth roots in a pre-digestion buffer containing 0.4 M EDTA (pH 8.0) and 16 µl/ml recombinant Proteinase K (Qiagen) and incubated at 37°C for 60 minutes at 900 rpm in a Thermomixer. The tooth roots were transferred to a digestion buffer containing 0.4 M EDTA (pH 8.0) and 16 µl/ml recombinant Proteinase K (Qiagen) and incubated at 37°C for 16 hours at 900 rpm in a Thermomixer. After incubation, the tooth roots were removed and wash with 70% EtOH and dried. The digestion buffers were transferred to ultrafiltration tubes (Amicon Ultra-4 Centrifugal Filter Unit 30K) with 3 mL TE (pH 8.0) and centrifuged at 2,000 g until the final volumes of 100 μL were obtained. These final volumes for each sample were then transferred to a silica column (MinElute PCR Purification Kit, QIAGEN) and purified following the manufacturer’s instructions with pre-heated (60°C) Tween 20 (a final concentration of 0.05%) added to 40 μl EB buffer at the final step.

#### DNA library construction and high-throughput sequencing.

Double-stranded DNA libraries were constructed using the NEBNext Ultra II DNA Library Prep Kit for Illumina (New England Biolabs) according to the manufacturer’s instructions. We assessed the quality of each library with a Bioanalyzer 2100 (Agilent). We verified the endogenous DNA content of each library through sequencing on the Illumina iSeq 100 platform. We used Illumina iSeq 100 reads to assess DNA damage patterns.

For genome sequencing, we treated the DNA with the USER (Uracil-Specific Excision Reagent) Enzyme (New England Biolabs) to remove uracil and reconstructed the DNA libraries. Paired-end sequencing (2 × 150 bp) was performed on the Illumina NovaSeq 6000 platform (S4 Table).

### Assessing DNA damage patterns

We used mapDamage2 [32] to assess DNA damage patterns in the ancient samples sequenced in this study, using default parameters. We used reads from Illumina iSeq 100 sequencing, non-uracil-removed libraries. All reads of the ancient samples showed C-to-T and G-to-A misincorporations at the 5’ and 3’ ends, respectively (S1 Fig).

#### Reads trimming and mapping.

We analyzed sequencing data from 152 modern and ancient dogs and gray wolves, and from six outgroup species (see S1 Table). The sequence reads were trimmed to eliminate nucleotides with an average base quality lower than 35 in 150 base pair reads (which corresponds to a cumulative base-calling error probability of less than 0.05 for a 150 base pair read), the length was less than 25 bp, and adaptor sequences were removed using CLC Genomics Workbench (https://www.qiagenbioinformatics.com/). We mapped the trimmed reads to the dog reference genome (CanFam3.1) using CLC Genomics Workbench under the following conditions: reads with high similarity (greater than 90% over more than 90% of their length) were mapped to the reference genome sequences to avoid including low-similarity reads. Additionally, any reads that mapped to multiple positions were removed using the “ignore” option to prevent mapping to non-unique regions. The mapping information of the ancient Korean dogs is shown in S4 Table.

Raw reads from 30 ancient dogs (coverage > 1x), 7 Japanese wolves, and 111 various modern dogs/wolves, along with 6 outgroup species, were downloaded from the NCBI Sequence Read Archive (SRA) or the European Nucleotide Archive (ENA) (S1 Table). Downloaded reads were analyzed in the same workflow as the sequenced samples in this study (CanFam 3.1).

#### Preparing gvcf file and genotyping a pseudo-haploid.

We exported the mapping data in BAM file format. The BAM files were sorted and indexed using SAMtools [33]. The duplicated reads in the BAM files were marked using MarkDuplicates algorithm, in GATK v4.2 (https://gatk.broadinstitute.org/hc/en-us). We performed genotype calling on all individuals analyzed in this study using the HaplotypeCaller algorithm in GATK v4.2. Genotypes for all individuals were output in the gvcf format (-ERC GVCF option). We combined all gvcf files into a single gvcf file using the CombineGVCFs algorithm in GATK v4.2 and filtered the combined file using VariantFiltration with default parameters. We used GenotypeGVCFs in GATK (ver.4.2) with the --ploidy setting option to obtain a pseudo-haploid vcf file.

### Phylogenetic analysis

To maximize the number of SNPs for analyses, we prepared four datasets for Neukdo1, Neukdo8, Neukdo20, and GB. We extracted the vcf data of two outgroup species, Coyote (*Canis latrans*) and Andean fox (*Lycalopex culpaeus*), six gray wolves including Japanese and Korean wolves, seven modern dogs, and five ancient dogs including one of the four ancient Korean dog (S1 Table) from the pseudo-haploid vcf file using bcftools [34]. We removed all sites with missing data and all indels using vcftools [35], as indels and missing data are not allowed in the phylogenetic analysis. Then, we removed all singleton and doubleton sites to eliminate PCR and sequencing errors using the minor allele count (mac) option (mac = 3) in vcftools [35]. We extracted sites with coverage of 1 or more in all individuals with GQ values of 5 or more using vcftools [35]. To eliminate the effect of DNA damage, we removed all transition sites. The final datasets consisted of 14,517 sites for the Neukdo1, 196,547 sites for the Neukdo8, 221,326 sites for the Neukdo20, and 76,216 sites for the GB vcf files.

We converted the vcf files for Neukdo1, Neukdo8, Neukdo20, and GB to PHYLIP format. 10-kb sequences from the 5’ end of the PHYLIP format file were used to select a model for the Maximum Likelihood method in MEGA ver. X [36]. We constructed phylogenetic trees for Neukdo1, Neukdo8, Neukdo20, and GB using the Maximum Likelihood (ML) method using PhyML ver. 3.2 [37] with a model selection option “-m GTR” and with 100 bootstrap replications

#### Principal component analysis.

We filtered the pseudo-haploid vcf file to remove sites with missingness higher than 30%, all indels, singleton and doubleton sites, and minor allele frequency less than 0.05 using vcftools [35]. We extracted sites with coverage of 1 or more in all individuals with GQ values of 5 or more using vcftools [35]. We also removed all transition sites to eliminate the effect of DNA damage. The final dataset consisted of 3,040,145 sites. Using this vcf file, we performed a principal component analysis (PCA) using PLINK ver. 1.9 [38] with an option “--indep-pairwise 50 10 0.1” to explore the affinity among all dogs/wolves (Figure 3a).

To clarify the affinity of the ancient Korean dogs and other dogs, we performed PCA using only dog individuals. For this analysis, we removed all wolves and outgroup species from the pseudo-haploid vcf file using bcftools [34]. Then we filter the dog-only vcf file to remove sites with missingness higher than 15%, all indels, singleton and doubleton sites, minor allele frequency less than 0.05, GQ values of 4 or less, and transition sites using vcftools [35]. The final dataset of a dog-only vcf dataset consisted of 302,355 sites. Using the dog-only vcf file, we performed a principal component analysis (PCA) using PLINK ver. 1.9 [38] with the same option (Figure 3b).

#### *f3*, *f4* statistics, and *f4*-ratio.

We use the same vcf file including 3,040,145 sites as the file for PCA of all individuals for the ADMIXTOOLS ver. 7.0.1 [39]. The vcf file was converted into eigenstrat format and used as an input file of ADMIXTOOLS. We used *f*3, *f4* statistics, and *f4*-ratio implemented in ADMIXTOOLS ver. 7.0.1 [39] according to the manual (https://github.com/DReichLab/AdmixTools?utm_source) to evaluate the shared genetic drift among dogs/wolves. For all analyses, we used Andean fox as an outgroup.

To measure shared genetic drift between ancient Korean dogs and other dogs, we used outgroup *f3* statistics. When we analyzed the *f3* statistics of ancient Korean dogs individually, their Z scores were often lower than 3, compared with those of other ancient dogs, due to the low number of available sites. Therefore, we used 4 ancient Korean dogs as a population. The *f3* values and the representative heat maps of the values are shown in Figs 4 and S2, respectively.

*f4* statistics testing the genetic affinity of the Neukdo1, the Neukdo8, the Neukdo20, and the GB with all other wolves are shown in S3–S6 Figs. The African dogs (African_Dog1, African_Dog2, African_Dog3, African_Dog4, African_Dog5, Basenji, Nigerian_Indigenous_Dog1, Nigerian_Indigenous_Dog2, Nigerian_Indigenous_Dog3, and Nigerian_Indigenous_Dog4) were used as an African dog population. In the *f4* statistics testing the genetic affinity of the European dogs with the East Eurasian dogs, the Ruropean dogs (Airedale_Terrier, American_Sta_Terrie, Boston_Terrier, Doberman_Pinscher, German_shepherd, Labrador_retriever1, Labrador_retriever2, Labrador_retriever3, Maltease, Miniature_Schnauzer, Portugal_Village_Dog1, Portugal_Village_Dog2, Scottish_Deerhounds, Standard_Poodle1, Standard_Poodle2, Yorkshire_Terrier) were used as a European dog population (S8 Fig). The tree topology and the testing genetic affinities between the dogs/wolves pairs are shown in each figure (S3–S6, and S8 Figs).

In the *f4*-ratio testing proportion of genome introgression from European dogs to East Eurasian dogs, each of the European dogs and African dogs were used as populations. The tree topology, the direction of the introgression, and the introgression pairs are shown in each Figures (S7 and S9 Figs).

## Supporting information

S1 Fig The frequency of misincorporation from C to T and G to A at the 3’ and 5’ ends of reads was determined without uracil removal. The x-axis represents nucleotide positions from 5’ and 3’ ends, and the y-axis represents the frequency of substitution for the dog reference genome (CanFam 3.1). The line in the left panel represents C to T mis-incorporation, and the line on the right represents G to A mis-incorporation.
(PDF)

S2 Fig Shared genetic drift between ancient Korean dogs and other dog breeds was analyzed using outgroup f3 statistics. The f3 statistical values are displayed in descending order, with the names of the dog breeds listed on the right side of the panel. Error bars indicate standard errors. The heatmap of the f3 values is shown in Fig 4.
(PDF)

S3 Fig The f4 statistics were used to test the genetic affinity of Neukdo1 in comparison to all other wolves. All African dogs were used as a population. f4 values for each combination are plotted. The f4 values are displayed in descending order, with the names of the wolves listed on the right side of the panel. f4 values with Z score over 3 are shown in blue. Error bars represent standard errors. *African_Dog1, African_Dog2, African_Dog3, African_Dog4, African_Dog5, Basenji, Nigerian_Indigenous_Dog1, Nigerian_Indigenous_Dog2, Nigerian_Indigenous_Dog3, and Nigerian_Indigenous_Dog4 were used as an African dog population.
(PDF)

S4 Fig The f4 statistics were used to test the genetic affinity of Neukdo8 in comparison to all other wolves. All African dogs were used as a population. f4 values for each combination are plotted. The f4 values are displayed in descending order, with the names of the wolves listed on the right side of the panel. f4 values with Z score over 3 are shown in blue. Error bars represent standard errors. *African_Dog1, African_Dog2, African_Dog3, African_Dog4, African_Dog5, Basenji, Nigerian_Indigenous_Dog1, Nigerian_Indigenous_Dog2, Nigerian_Indigenous_Dog3, and Nigerian_Indigenous_Dog4 were used as an African dog population.
(PDF)

S5 Fig The f4 statistics were used to test the genetic affinity of Neukdo20 in comparison to all other wolves. All African dogs were used as a population. f4 values for each combination are plotted. The f4 values are displayed in descending order, with the names of the wolves listed on the right side of the panel. f4 values with Z score over 3 are shown in blue. Error bars represent standard errors. *African_Dog1, African_Dog2, African_Dog3, African_Dog4, African_Dog5, Basenji, Nigerian_Indigenous_Dog1, Nigerian_Indigenous_Dog2, Nigerian_Indigenous_Dog3, and Nigerian_Indigenous_Dog4 were used as an African dog population.
(PDF)

S6 Fig The f4 statistics were used to test the genetic affinity of GB in comparison to all other wolves. All African dogs were used as a population. f4 values for each combination are plotted. The f4 values are displayed in descending order, with the names of the wolves listed on the right side of the panel. f4 values with Z score over 3 are shown in blue. Error bars represent standard errors. *African_Dog1, African_Dog2, African_Dog3, African_Dog4, African_Dog5, Basenji, Nigerian_Indigenous_Dog1, Nigerian_Indigenous_Dog2, Nigerian_Indigenous_Dog3, and Nigerian_Indigenous_Dog4 were used as an African dog population.
(PDF)

S7 Fig The f4-ratio test estimates the proportion of genome introgression from the Japanese wolf to dogs. Each f4-ratio α value is plotted in ascending order, with the names of the dog breeds displayed on the right side of the panel. Error bars indicate standard errors.
(PDF)

S8 Fig The f4 statistics were used to test the genetic affinity of the European dogs with the East Eurasian dogs. f4 values for each combination are plotted. The f4 values are displayed in descending order, with the names of the dogs are shown on the right side of the panel. f4 values with Z score over 3 are shown in blue. Error bars represent standard errors. *African_Dog1, African_Dog2, African_Dog3, African_Dog4, African_Dog5, Basenji, Nigerian_Indigenous_Dog1, Nigerian_Indigenous_Dog2, Nigerian_Indigenous_Dog3, and Nigerian_Indigenous_Dog4 were used as a African dog population. **Airedale_Terrier, American_Sta_Terrie, Boston_Terrier, Doberman_Pinscher, German_shepherd, Labrador_retriever1, Labrador_retriever2, Labrador_retriever3, Maltease, Miniature_Schnauzer, Portugal_Village_Dog1, Portugal_Village_Dog2, Scottish_Deerhounds, Standard_Poodle1, Standard_Poodle2, Yorkshire_Terrier were used as a European dog population.
(PDF)

S9 Fig The f4-ratio test estimates the proportion of genome introgression from European dogs to East Eurasian dogs. Each f4-ratio α value is plotted in ascending order, with the names of the dogs are shown on the right side of the panel. Error bars represent standard errors. *African_Dog1, African_Dog2, African_Dog3, African_Dog4, African_Dog5, Basenji, Nigerian_Indigenous_Dog1, Nigerian_Indigenous_Dog2, Nigerian_Indigenous_Dog3, and Nigerian_Indigenous_Dog4 were used as a African dog population. **Airedale_Terrier, American_Sta_Terrie, Boston_Terrier, Doberman_Pinscher, German_shepherd, Labrador_retriever1, Labrador_retriever2, Labrador_retriever3, Maltease, Miniature_Schnauzer, Portugal_Village_Dog1, Portugal_Village_Dog2, Scottish_Deerhounds, Standard_Poodle1, Standard_Poodle2, Yorkshire_Terrier were used as a European dog population.
(PDF)

S1 Table Sample information. (XLSX)

S2 Table Provenance, repository, and identification details of archaeological dog specimens used in this study. (XLSX)

S3 Table Radiocarbon Dates from the Bonghwang-dong and Neukdo Sites. (XLSX)

S4 Table Mapping information. (XLSX)
