# Supplementary material for: Ancient East Asian dog lineage is revealed by genome of ancient Korean dogs
Source: PLoS One. 2026 May 6;21(5):e0346864. doi: 10.1371/journal.pone.0346864 (PMC13148662; doi:10.1371/journal.pone.0346864)
Supplement: S1 Fig — The x-axis represents nucleotide positions from 5’ and 3’ ends, and the y-axis represents the frequency of substitution for the dog reference genome (CanFam 3.1). The line in the left panel represents C to T mis-incorporation, and the line on the right represents G to A mis-incorporation. (PDF) [file pone.0346864.s001.pdf]

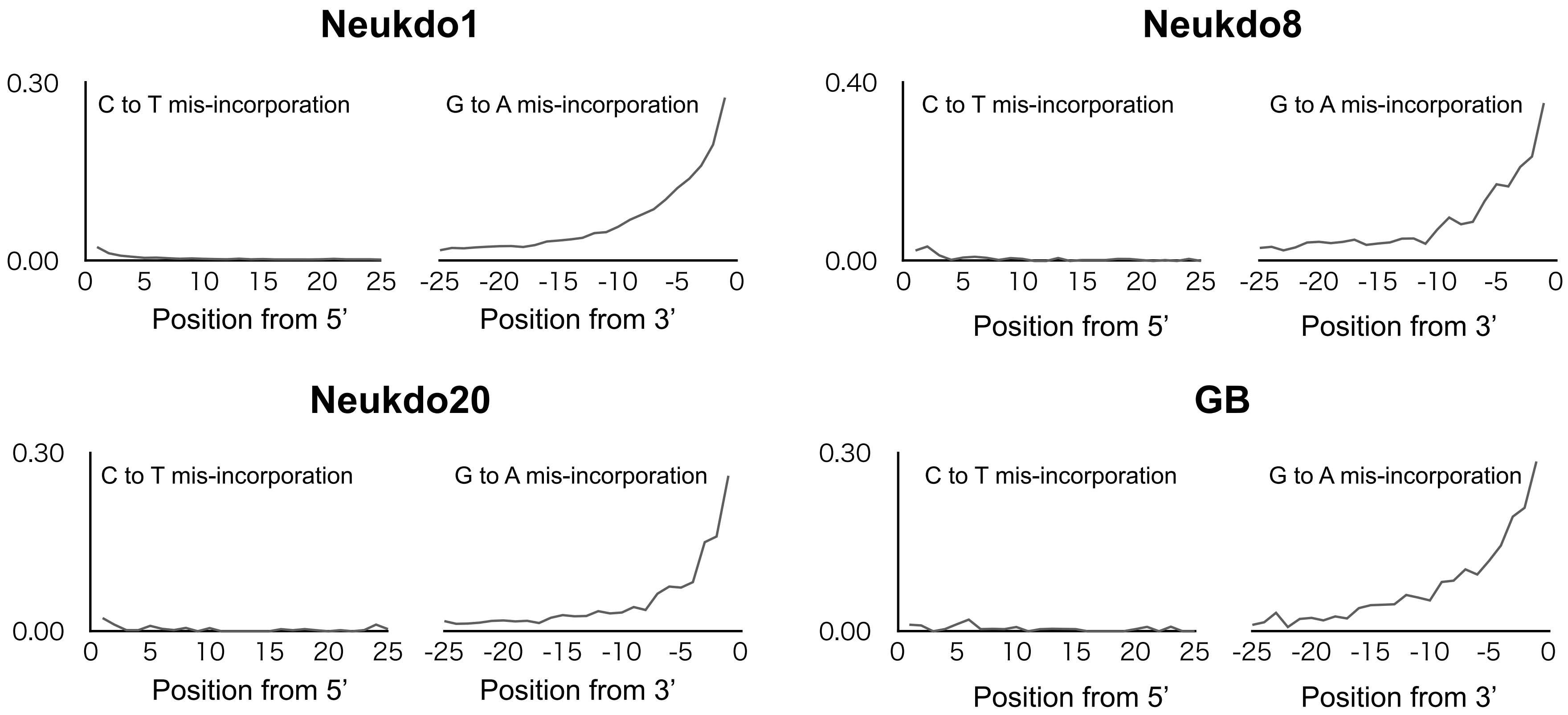

Figure S1

The frequency of misincorporation from C to T and G to A at the 3’ and 5’ ends of reads was determined without uracil removal. The x-axis represents nucleotide positions from 5' and 3' ends, and the y-axis represents the frequency of substitution for the dog reference genome (CanFam 3.1). The line in the left panel represents C to T mis-incorporation, and the line on the right represents G to A mis-incorporation.
