# Supplementary material for: Ancient East Asian dog lineage is revealed by genome of ancient Korean dogs
Source: PLoS One. 2026 May 6;21(5):e0346864. doi: 10.1371/journal.pone.0346864 (PMC13148662; doi:10.1371/journal.pone.0346864)
Supplement: S2 Fig — The f3 statistical values are displayed in descending order, with the names of the dog breeds listed on the right side of the panel. Error bars indicate standard errors. The heatmap of the f3 values is shown in Fig 4. (PDF) [file pone.0346864.s002.pdf]

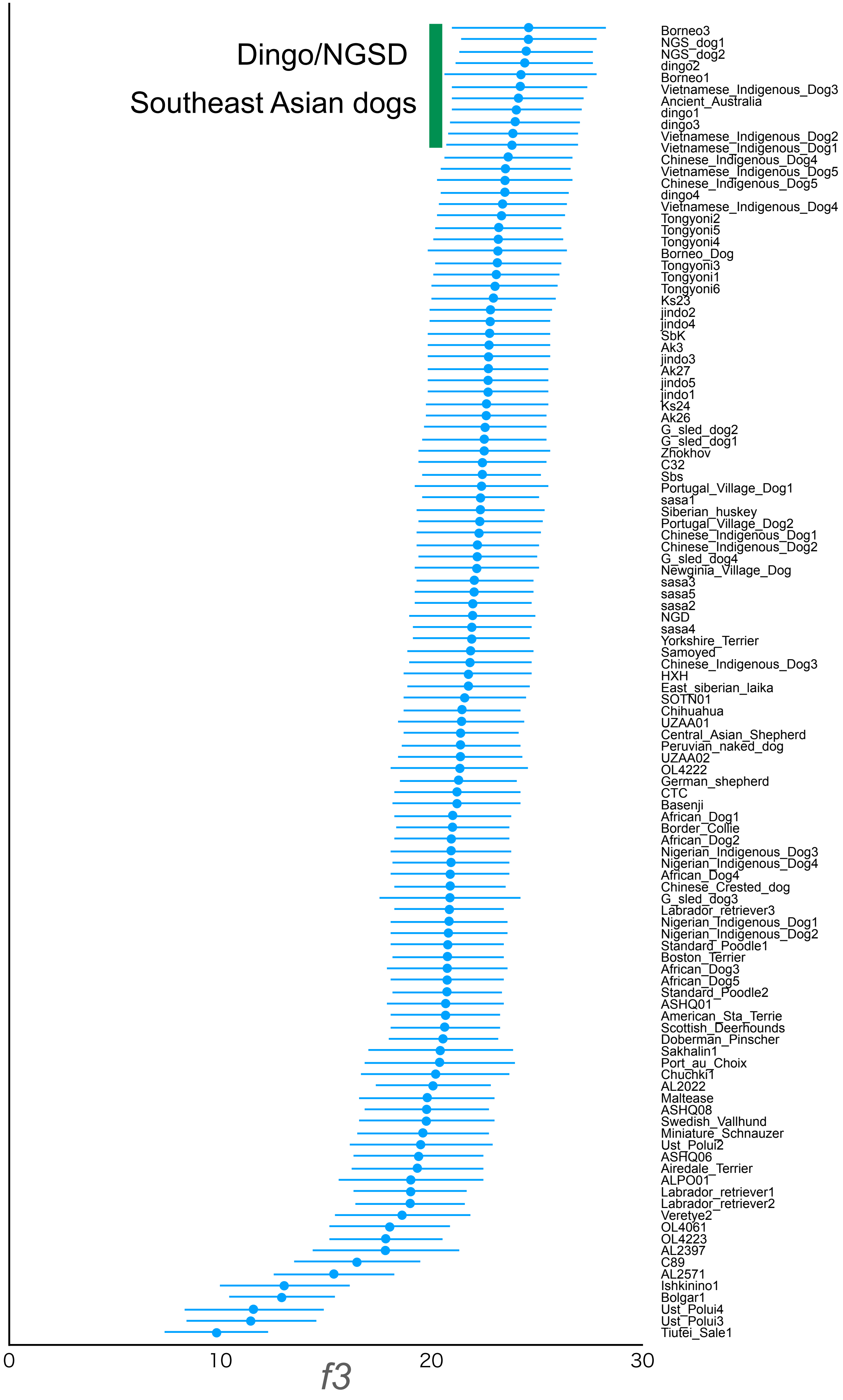

Figure S2
Shared genetic drift between ancient Korean dogs and other dog breeds was analyzed using outgroup f3 statistics. The f3 statistical values are displayed in descending order, with the names of the dog breeds listed on the right side of the panel. Error bars indicate standard errors. The heatmap of the f3 values is shown in Figure 4.
