# Supplementary material for: Ancient East Asian dog lineage is revealed by genome of ancient Korean dogs
Source: PLoS One. 2026 May 6;21(5):e0346864. doi: 10.1371/journal.pone.0346864 (PMC13148662; doi:10.1371/journal.pone.0346864)
Supplement: S6 Fig — All African dogs were used as a population. f4 values for each combination are plotted. The f4 values are displayed in descending order, with the names of the wolves listed on the right side of the panel. f4 values with Z score over 3 are shown in blue. Error bars represent standard errors. *African_Dog1, African_Dog2, African_Dog3, African_Dog4, African_Dog5, Basenji, Nigerian_Indigenous_Dog1, Nigerian_Indigenous_Dog2, Nigerian_Indigenous_Dog3, and Nigerian_Indigenous_Dog4 were used as an African dog population. (PDF) [file pone.0346864.s006.pdf]

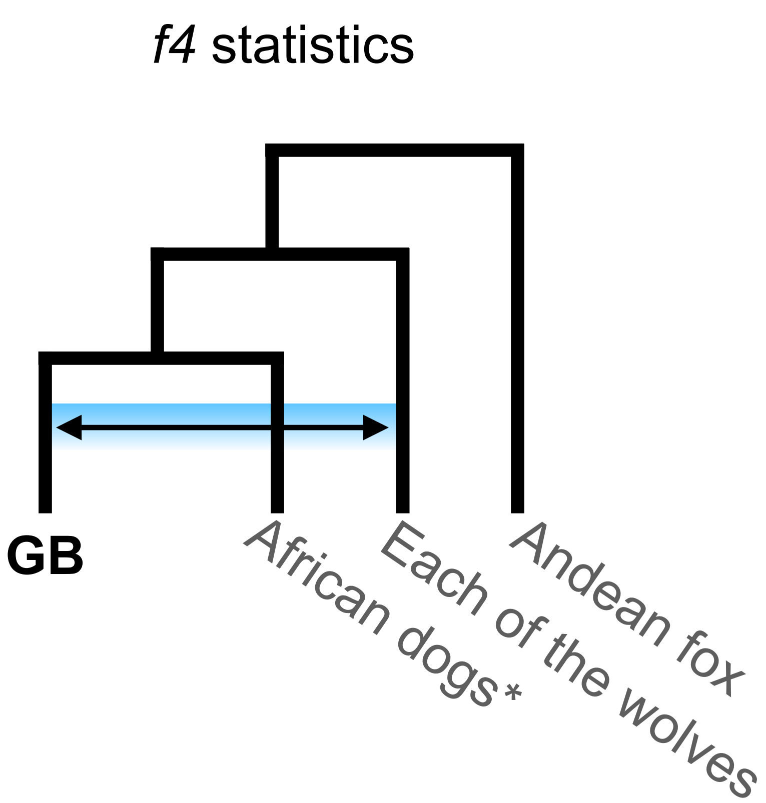

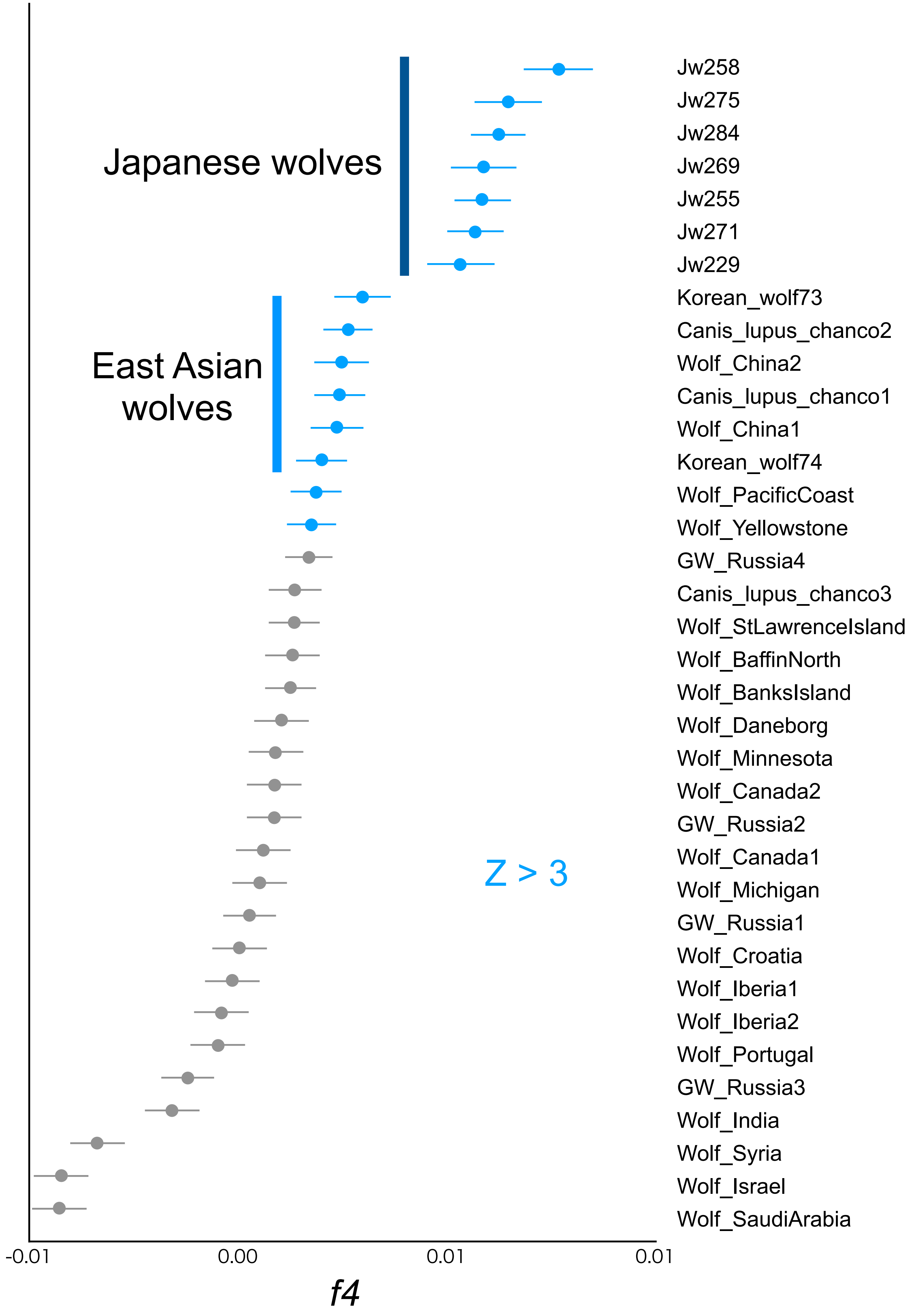

Figure S6
The *f4* statistics were used to test the genetic affinity of GB in comparison to all other wolves. All African dogs were used as a population. *f4* values for each combination are plotted. The *f4* values are displayed in descending order, with the names of the wolves listed on the right side of the panel. *f4* values with Z score over 3 are shown in blue. Error bars represent standard errors.
*African_Dog1, African_Dog2, African_Dog3, African_Dog4, African_Dog5, Basenji, Nigerian_Indigenous_Dog1, Nigerian_Indigenous_Dog2, Nigerian_Indigenous_Dog3, and Nigerian_Indigenous_Dog4 were used as an African dog population.
