# Supplementary material for: Ancient East Asian dog lineage is revealed by genome of ancient Korean dogs
Source: PLoS One. 2026 May 6;21(5):e0346864. doi: 10.1371/journal.pone.0346864 (PMC13148662; doi:10.1371/journal.pone.0346864)
Supplement: S7 Fig — Each f4-ratio α value is plotted in ascending order, with the names of the dog breeds displayed on the right side of the panel. Error bars indicate standard errors. (PDF) [file pone.0346864.s007.pdf]

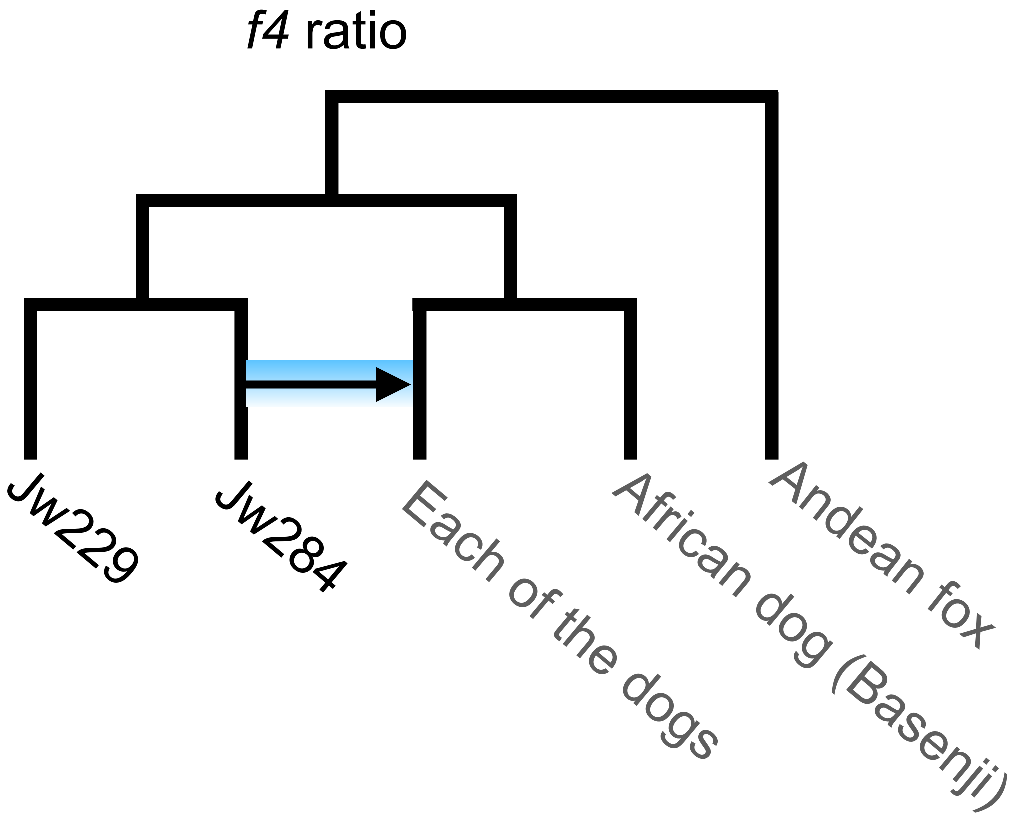

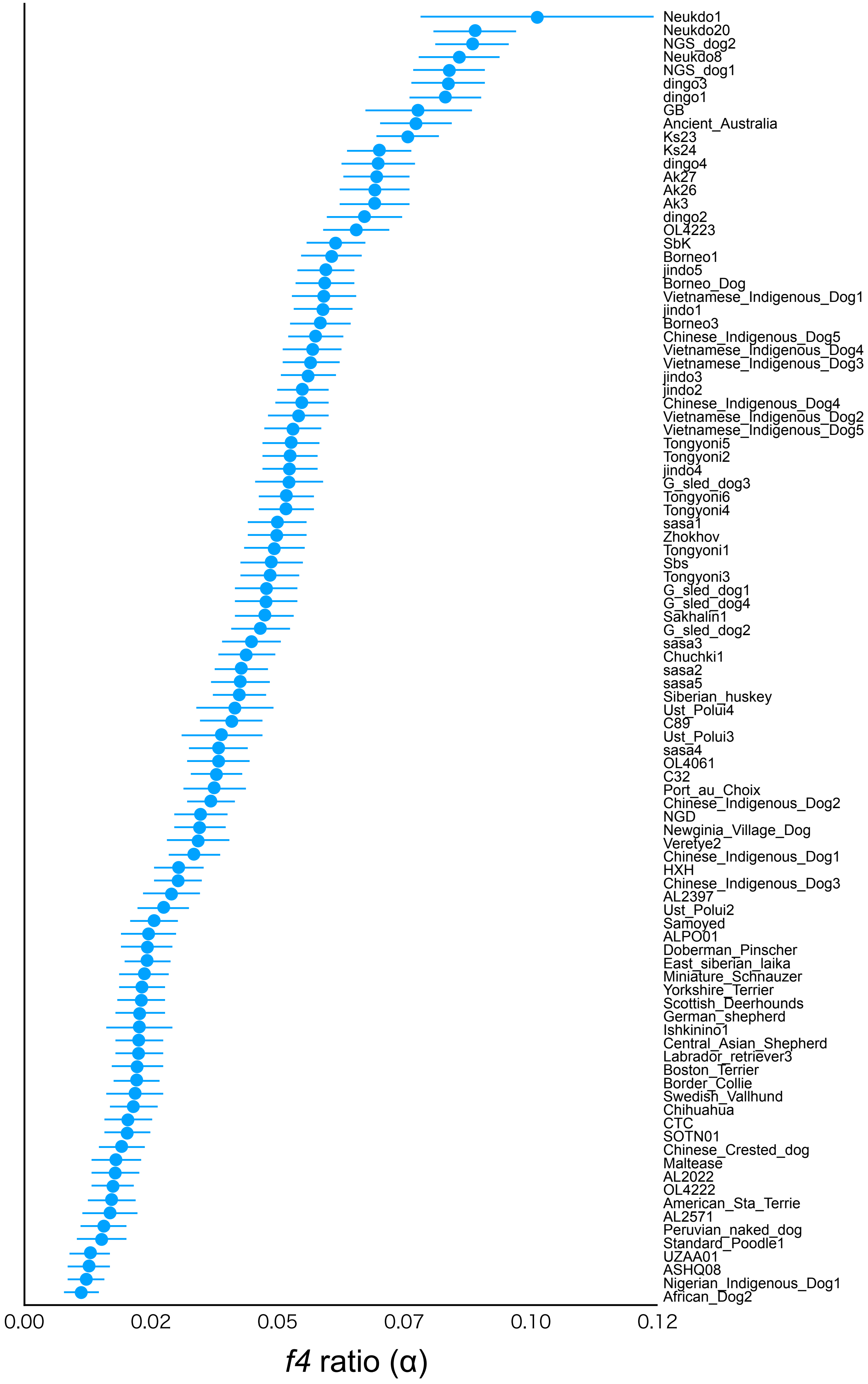

Figure S7
The *f4*-ratio test estimates the proportion of genome introgression from the Japanese wolf to dogs. Each *f4*-ratio α value is plotted in ascending order, with the names of the dog breeds displayed on the right side of the panel. Error bars indicate standard errors.
