# Supplementary material for: Ancient East Asian dog lineage is revealed by genome of ancient Korean dogs
Source: PLoS One. 2026 May 6;21(5):e0346864. doi: 10.1371/journal.pone.0346864 (PMC13148662; doi:10.1371/journal.pone.0346864)
Supplement: S8 Fig — f4 values for each combination are plotted. The f4 values are displayed in descending order, with the names of the dogs are shown on the right side of the panel. f4 values with Z score over 3 are shown in blue. Error bars represent standard errors. *African_Dog1, African_Dog2, African_Dog3, African_Dog4, African_Dog5, Basenji, Nigerian_Indigenous_Dog1, Nigerian_Indigenous_Dog2, Nigerian_Indigenous_Dog3, and Nigerian_Indigenous_Dog4 were used as a African dog population. **Airedale_Terrier, American_Sta_Terrie, Boston_Terrier, Doberman_Pinscher, German_shepherd, Labrador_retriever1, Labrador_retriever2, Labrador_retriever3, Maltease, Miniature_Schnauzer, Portugal_Village_Dog1, Portugal_Village_Dog2, Scottish_Deerhounds, Standard_Poodle1, Standard_Poodle2, Yorkshire_Terrier were used as a European dog population. (PDF) [file pone.0346864.s008.pdf]

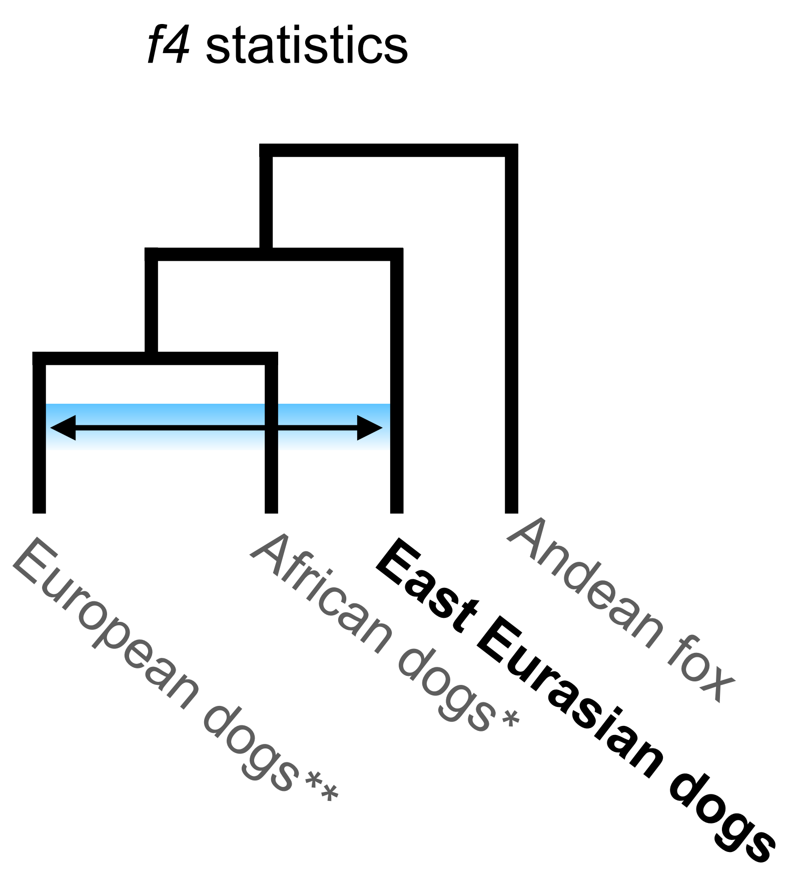

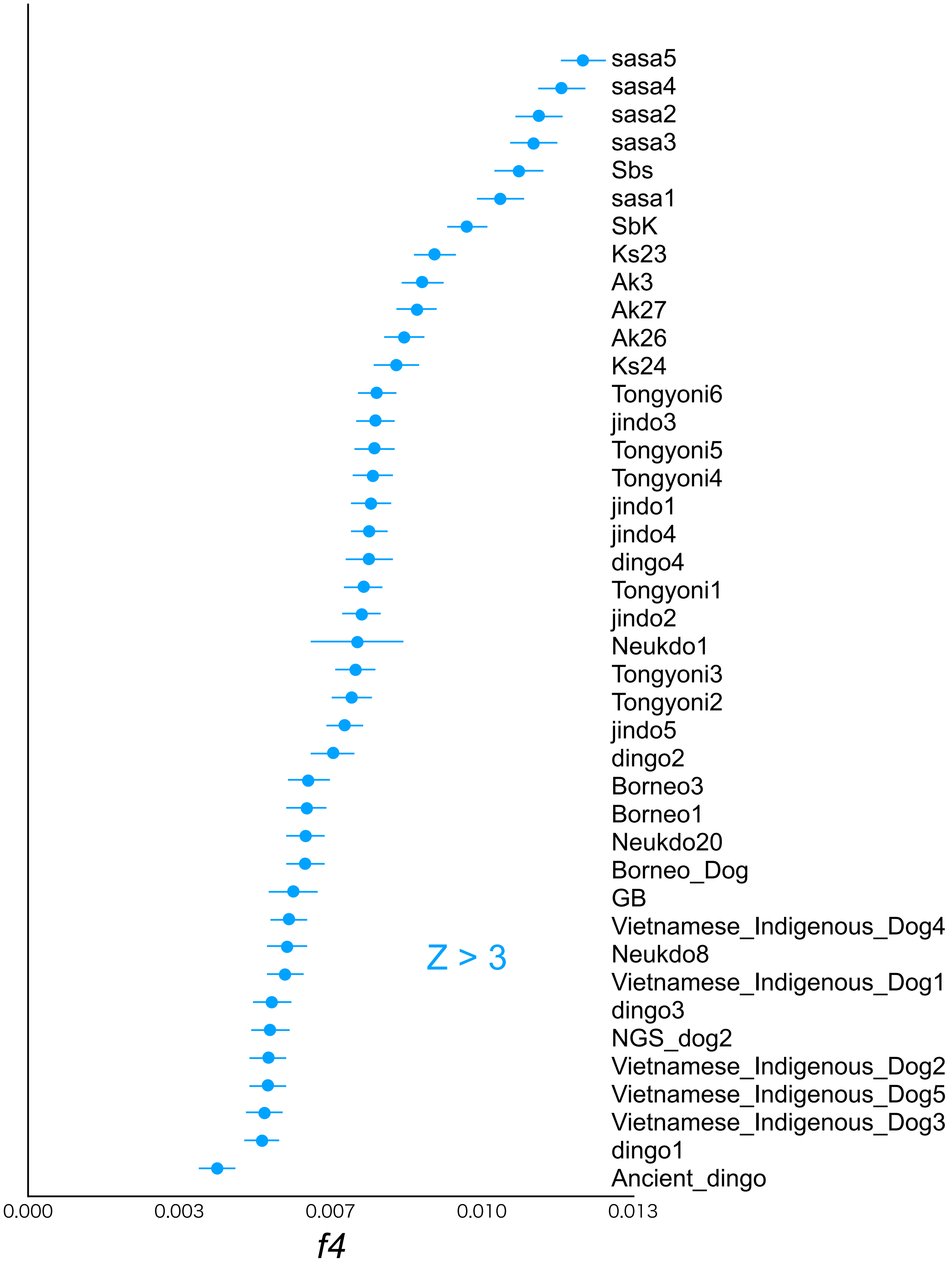

Figure S8
The *f4* statistics were used to test the genetic affinity of the European dogs with the East Eurasian dogs. *f4* values for each combination are plotted. The *f4* values are displayed in descending order, with the names of the dogs are shown on the right side of the panel. *f4* values with Z score over 3 are shown in blue. Error bars represent standard errors.
*African_Dog1, African_Dog2, African_Dog3, African_Dog4, African_Dog5, Basenji, Nigerian_Indigenous_Dog1, Nigerian_Indigenous_Dog2, Nigerian_Indigenous_Dog3, and Nigerian_Indigenous_Dog4 were used as a African dog population.
**Airedale_Terrier, American_Sta_Terrie, Boston_Terrier, Doberman_Pinscher, German_shepherd, Labrador_retriever1, Labrador_retriever2, Labrador_retriever3, Maltease, Miniature_Schnauzer, Portugal_Village_Dog1, Portugal_Village_Dog2, Scottish_Deerhounds, Standard_Poodle1, Standard_Poodle2, Yorkshire_Terrier were used as a European dog population.
