# Supplementary material for: Ancient East Asian dog lineage is revealed by genome of ancient Korean dogs
Source: PLoS One. 2026 May 6;21(5):e0346864. doi: 10.1371/journal.pone.0346864 (PMC13148662; doi:10.1371/journal.pone.0346864)
Supplement: S9 Fig — Each f4-ratio α value is plotted in ascending order, with the names of the dogs are shown on the right side of the panel. Error bars represent standard errors. *African_Dog1, African_Dog2, African_Dog3, African_Dog4, African_Dog5, Basenji, Nigerian_Indigenous_Dog1, Nigerian_Indigenous_Dog2, Nigerian_Indigenous_Dog3, and Nigerian_Indigenous_Dog4 were used as a African dog population. **Airedale_Terrier, American_Sta_Terrie, Boston_Terrier, Doberman_Pinscher, German_shepherd, Labrador_retriever1, Labrador_retriever2, Labrador_retriever3, Maltease, Miniature_Schnauzer, Portugal_Village_Dog1, Portugal_Village_Dog2, Scottish_Deerhounds, Standard_Poodle1, Standard_Poodle2, Yorkshire_Terrier were used as a European dog population. (PDF) [file pone.0346864.s009.pdf]

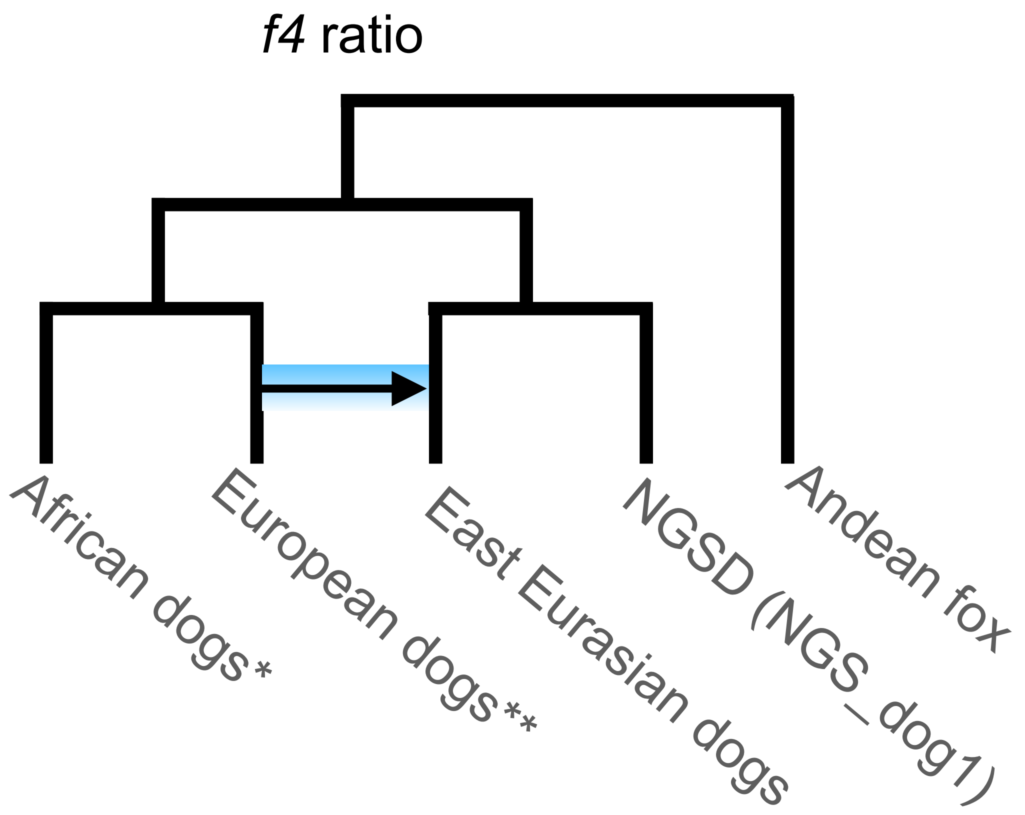

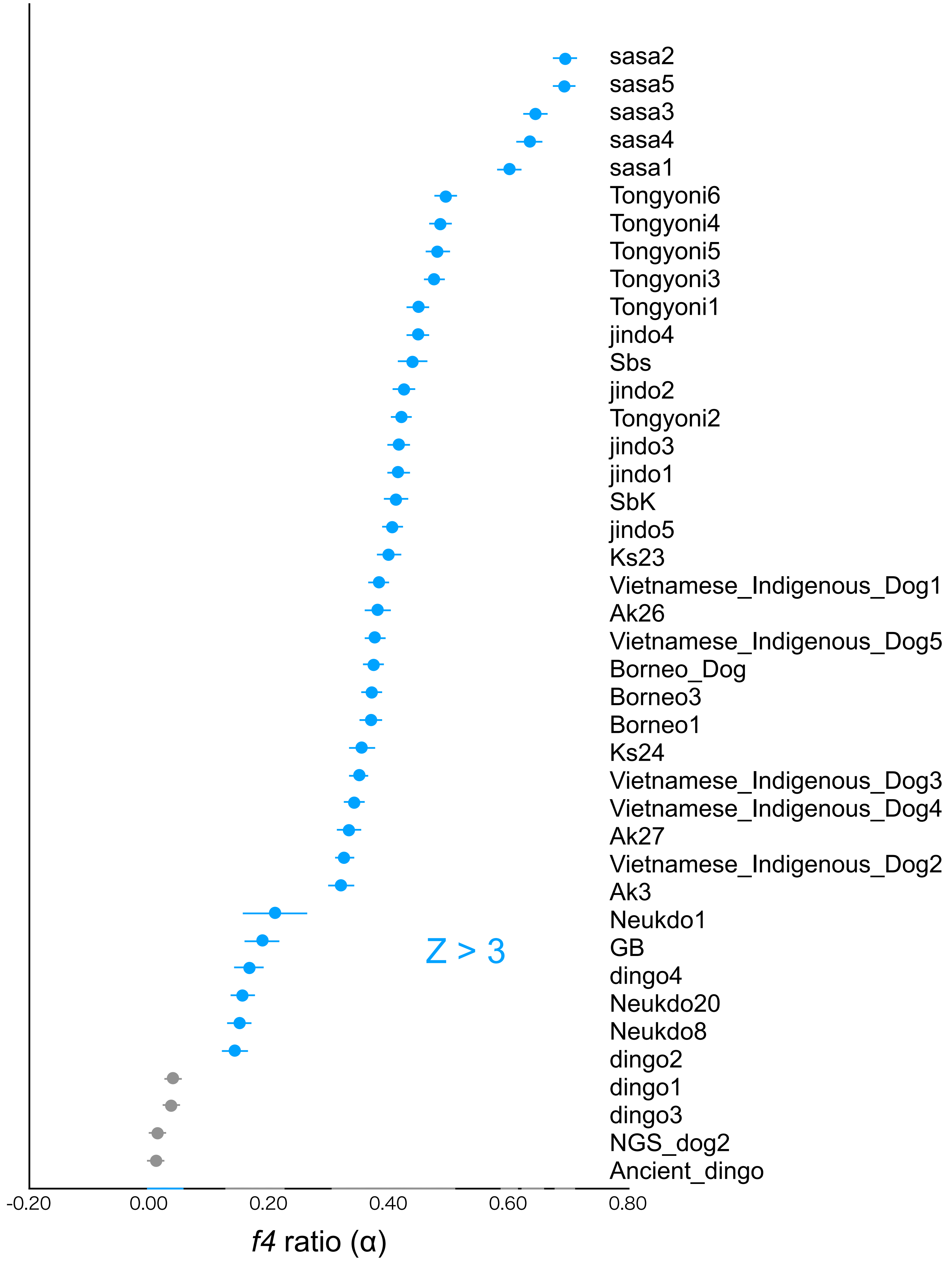

Figure S9
The *f4*-ratio test estimates the proportion of genome introgression from European dogs to East Eurasian dogs. Each *f4*-ratio α value is plotted in ascending order, with the names of the dogs are shown on the right side of the panel. Error bars represent standard errors.
*African_Dog1, African_Dog2, African_Dog3, African_Dog4, African_Dog5, Basenji, Nigerian_Indigenous_Dog1, Nigerian_Indigenous_Dog2, Nigerian_Indigenous_Dog3, and Nigerian_Indigenous_Dog4 were used as a African dog population.
**Airedale_Terrier, American_Sta_Terrie, Boston_Terrier, Doberman_Pinscher, German_shepherd, Labrador_retriever1, Labrador_retriever2, Labrador_retriever3, Maltease, Miniature_Schnauzer, Portugal_Village_Dog1, Portugal_Village_Dog2, Scottish_Deerhounds, Standard_Poodle1, Standard_Poodle2, Yorkshire_Terrier were used as a European dog population.
